# An overview of mutational and copy number signatures in human cancer

**DOI:** 10.1002/path.5912

**Published:** 2022-05-20

**Authors:** Christopher D Steele, Nischalan Pillay, Ludmil B Alexandrov

**Affiliations:** ^1^ Research Department of Pathology, Cancer Institute University College London London UK; ^2^ Department of Cellular and Molecular Pathology Royal National Orthopaedic Hospital NHS Trust Stanmore UK; ^3^ Department of Cellular and Molecular Medicine, UC San Diego La Jolla CA USA; ^4^ Department of Bioengineering, UC San Diego La Jolla CA USA; ^5^ Moores Cancer Center, UC San Diego La Jolla CA USA

**Keywords:** mutational signatures, copy number signatures, genomics, cancer

## Abstract

The genome of each cell in the human body is constantly under assault from a plethora of exogenous and endogenous processes that can damage DNA. If not successfully repaired, DNA damage generally becomes permanently imprinted in cells, and all their progenies, as somatic mutations. In most cases, the patterns of these somatic mutations contain the tell‐tale signs of the mutagenic processes that have imprinted and are termed mutational signatures. Recent pan‐cancer genomic analyses have elucidated the compendium of mutational signatures for all types of small mutational events, including (1) single base substitutions, (2) doublet base substitutions, and (3) small insertions/deletions. In contrast to small mutational events, where, in most cases, DNA damage is a prerequisite, aneuploidy, which refers to the abnormal number of chromosomes in a cell, usually develops from mistakes during DNA replication. Such mistakes include DNA replication stress, mitotic errors caused by faulty microtubule dynamics, or cohesion defects that contribute to chromosomal breakage and can lead to copy number (CN) alterations (CNAs) or even to structural rearrangements. These aberrations also leave behind genomic scars which can be inferred from sequencing as CN signatures and rearrangement signatures. The analyses of mutational signatures of small mutational events have been extensively reviewed, so we will not comprehensively re‐examine them here. Rather, our focus will be on summarising the existing knowledge for mutational signatures of CNAs. As studying CN signatures is an emerging field, we briefly summarise the utility that mutational signatures of small mutational events have provided in basic science, cancer treatment, and cancer prevention, and we emphasise the future role that CN signatures may play in each of these fields. © 2022 The Authors. *The Journal of Pathology* published by John Wiley & Sons Ltd on behalf of The Pathological Society of Great Britain and Ireland.

## The difference between DNA damage and somatic mutations

Some processes, mainly those due to internal cellular mechanisms, are ubiquitous and can be found in every cell. Examples of such ubiquitous processes include generation of reactive oxygen species, as part of the normal functioning of the mitochondria, or internal cellular processes inadvertently mutating DNA during replication [1]. Other processes are tissue‐specific or can be due to lifestyle choices or to environmental exposures. For example, ultraviolet radiation (UV) in sunlight will damage the DNA of skin cells, but it will not affect cells in internal organs (e.g. cells in the liver or pancreas). Similarly, smoking tobacco cigarettes will damage the DNA of certain organs, most prominently cells of the lung, but it will not alter the genomes of cells in other organs, such as the brain. These endogenous and exogenous processes result in DNA damage. In general, DNA damage refers to *chemical modifications* of nucleotides (adenine, thymine, guanine, and cytosine) as well as to changes/breaks in the covalent bonds between adjacent nucleotides or the hydrogen bonds between traditional DNA base‐pairs. Some examples of DNA damage are oxidation, deamination, and alkylation of DNA bases; formation of single‐ and double‐strand breaks; formation of covalent linkages between consecutive bases along the nucleotide chain; and many others [2]. In contrast, somatic mutations reflect a *change in the sequence* of the base‐pair content in DNA. Somatic mutations could include small mutational events such as substitutions, where a DNA base‐pair is substituted with another DNA base‐pair (e.g. C:G base‐pair changes to a T:A base‐pair), and small insertions/deletions, where, for example, a set of DNA base‐pairs is completely removed from the genome (e.g. a set of CAC:GTG base‐pairs gets deleted from the genome). Somatic mutations can also include large mutational events affecting many millions of base pairs across multiple chromosomes. Examples of such types of mutations generally include genomic structural variants (SVs), where, for example, two chromosomes can become partially fused, or copy number (CN) variations, where the number of copies of a large genomic segment gets amplified or deleted. Some examples of CN changes are a loss of a chromosome arm, doubling the number of copies of a specific chromosome, and even the entire diploid genome: doubling the number of copies (whole‐genome duplication) or losing a copy of the diploid genome (haploidisation).

An important difference between *damaged* and *mutated* DNA is that while some DNA damage may persist for decades, it is generally confined to the cell in which it occurred [3]. In contrast, somatic mutations affect both their cell of origin and all progenitors of that cell, thus having an exponential functional effect in all offspring. While, in many cases, somatic mutations require pre‐existing DNA damage, the road from a damaged DNA to a somatic mutation involves multiple additional molecular steps – with the vast majority of DNA damage being repaired and not resulting in any somatic mutations. However, a failure of a DNA repair pathway in a cell can result in a specific type of DNA damage being un‐ or under‐repaired, causing a mutator phenotype, and resulting in an explosion of somatic mutations, e.g. mismatch repair deficiency [4]. Importantly, not all mutations necessitate prior DNA damage; examples include infidelity of the polymerases during replication, leading to substitutions or indels in progenitor cells, as well as large CN events such whole‐genome doubling caused through mitotic error [5].

In most cases, sequencing the genomes of cancer or normal somatic cells allows the derivation of somatic mutations, but it does not directly allow the study of DNA damage. Nevertheless, as the activity of DNA damage and repair processes reflects many of the imprinted mutations, analysis of somatic mutations may also be leveraged to understand DNA damage and repair processes. Different types of sequencing approaches provide different resolutions in examining different types of somatic mutations [6]. A complete sequencing of a genome (also known as whole‐genome sequencing, WGS) allows examination for all types of somatic mutations, which include (1) single base substitutions (SBSs); (2) small indels and deletions (indels); (3) copy number alterations (CNAs); and (4) genomic SVs. In contrast, sequencing of all exons of the genome, also known as whole‐exome sequencing (WES), allows interrogation for substitutions and indels in coding regions as well as providing limited resolution of genome‐wide CNAs. Sequencing only a small set of actionable genes, such the targeted panels commonly used for cancer diagnosis, can be utilised to detect substitutions and indels in these genes and may provide an extremely low‐resolution map for detecting certain very large genomic segments with CNAs. In most cases, whole‐exome and targeted‐panel sequencing do not allow detection of SVs, unless the experimental protocols have been designed to detect specific SVs.

## Understanding somatic mutagenesis through mutational signatures

Somatic mutations have been shown to play a pivotal role in cancer initiation and cancer evolution [7, 8]. Mutations that impact gene functionality, either through a gain or through a loss of function, and enhance the fitness characteristics of a cell are often referred to as *driver mutations*. While non‐neoplastic and cancer cells harbour fewer than ten driver mutations, their genomes are moulded with many thousands (and, in some cases, even millions) of somatic mutations that provide little fitness advantage; such mutations are generally termed *passenger mutations* [9]. The distribution of passenger mutations across the genomic landscape can be affected by several different factors that alter DNA damage, DNA repair, or other cellular processes. From a genomic architecture perspective, the locations of passenger mutations are modified by replication timing, transcriptional activity, eu‐ and hetero‐chromatin, histone modifications, transcription factor binding sites, and factors related to nucleosome occupancy [10]. From a local sequencing context perspective, different mutational processes have different biophysical and biochemical characteristics, resulting in a specific preference for the immediate sequence context of the imprinted somatic mutations. This combination of micro‐ and macro‐genomic features affects the accumulation of somatic mutations from a particular mutational process, leading to a characteristic pattern of somatic mutations, termed the *mutational signature*.

The first descriptions of specific patterns of DNA damage identified through exogenous sources were presented in the late 1950s, when it was discovered that UV light exposure of *in vitro* systems resulted in the formation of pyrimidine photodimers (two consecutive bases on one strand that bind together), predominantly affecting adjacent cytosine and thymine nucleotides and leading to cytosine–cytosine, cytosine–thymine, thymine–cytosine, and thymine–thymine photodimers [11, 12, 13]. Subsequent work described the biophysical and biochemical properties leading to the formation of DNA damage from UV light, including both cyclobutane pyrimidine dimers (CPDs) and pyrimidine(6‐4)pyrimidone photoproducts. Almost 20 years later, with the advent of Sanger sequencing, a causative molecular link was made between UV‐induced DNA damage, subsequent formation of somatic mutations, and the development of skin cancer by examining the patterns of mutations in certain skin cancers [14]. Similar research was also performed for a number of other cancer types by evaluating the patterns of mutations in the exons of *TP53*, the most commonly mutated gene in human cancer, revealing a number of distinct mutational patterns including ones attributed to endogenous deamination, tobacco smoking, aflatoxin, and others.

The advances in next‐generation sequencing technologies have facilitated the unbiased assessment of the mutational patterns of cancer genomes [15, 16]. By sequencing the genome of a cancer, one can now observe the combined outcome of all mutational forces that have been active at different strengths and at different times throughout the lineage of the cancer cell. Importantly, by utilising a proper mathematical model and a set of machine learning (ML) computational approaches, one can decipher the individual signature of each process that has been active throughout this lineage. Moreover, by uniting the ML‐derived mutational signatures with experimental data, one can even understand the mutational processes that gave rise to these signatures and that caused the mutations in a cancer patient [17].

During the last decade, analysis of mutational signatures has become a standard approach in examining somatic mutations derived from next‐generation sequencing data (see [2, 18, 19] for in depth reviews of mutational signatures). In the majority of cases, these analyses have utilised somatic mutations derived from whole‐exome and/or whole‐genome sequenced cancer genomes by applying unsupervised ML approaches based on non‐negative matrix factorisation (NMF). In brief, from an analysis perspective, the mutations in a set of cancer genomes are categorised based on a mutational classification into distinct categories resulting into a mutational matrix, where each column reflects a cancer genome, each row a distinct mutation type, and the value of each cell corresponds to the number of mutations of a particular mutation type in a cancer genome. The mutational matrix is subsequently factorised with NMF into two matrices, one reflecting the mutational signatures and the other corresponding to the activities of each signature in each sample. Initially, analysis of mutational signatures across cancer types was performed only for SBSs, due to the simplicity in classifying these types of mutations, but has now expanded to include SBSs, doublet base substitutions (DBSs), small insertions and deletions (IDs), SVs, and CN signatures [20, 21, 22, 23, 24].

## Biological underpinnings and clinical implications of mutational signatures

From a research perspective, mutational signatures of small mutational events have provided a novel toolset for indirectly studying the molecular processes of DNA damage, DNA repair, and even DNA replication. Analyses of cancer genomes have elucidated the effect of genome architecture and the topographical features of the human genome on the cancer‐specific accumulation of somatic mutations from some mutational signatures [19]. Experimental works have also revealed the compendium of *in vitro*‐induced mutational signatures and the vulnerabilities of human stem cells to different endogenous and exogenous mutagens. Importantly, examination of mutational signatures has brought significant insights into the interactions between mismatch repair and replication; enzymatic deamination by AID/APOBEC3; transcription‐coupled repair; transcription‐coupled damage; topoisomerases and DNA repair; clustered mutagenesis and genomic rearrangements; and many others [25, 26, 27, 28, 29, 30, 31].

From a cancer prevention perspective, analysis of SBS, DBS, and ID mutational signatures has revealed a number of environmental mutagens causing specific cancer types with notable examples including (1) aristolochic acid: a group of acids found naturally in many types of plants, which has been linked to cancers of the liver, bladder, kidney, oral cavities, and oesophagus; (2) aflatoxin: a family of toxins produced by certain fungi, linked to liver cancer; (3) colibactin: a potent genotoxin associated with certain strains of *Escherichia coli*, found in colorectal cancer; and many others [32, 33, 34, 35]. These findings have allowed the proposal and development of strategies for preventing cancer by limiting exposure to such mutagens. Additionally, mutational signatures have been used as multi‐tumour phenotypes of germline predisposition, which has allowed for better screening of people with a higher risk for developing cancer. A notable example is that of *NTHL1*, where germline mutations can give rise to a multitude of different cancer types, which, prior to mutational signatures, was not fully appreciated [36].

From a cancer treatment perspective, mutational signatures have proven to be a valuable resource both for understanding iatrogenic exposures leading to secondary/recurrent cancers and for optimally targeting cancer therapies. Several studies have revealed the mutational signatures of chemo‐ and immune‐therapies, demonstrating, in many cases, that secondary cancers are caused by specific treatment regimens. Notable examples include secondary cancers after treatment with temozolomide and platinum therapies, as well as the role of azathioprine – a drug used in auto‐immune conditions – in causing primary cancers. Importantly, mutational signatures have also been shown to have clinical utility in selecting an optimum treatment strategy. A presence of specific mutational signatures has been used as a predictive biomarker for response to PARP inhibitors, platinum therapy, immunotherapy, and tamoxifen resistance [37, 38, 39].

Large‐scale national and international cancer genome sequencing efforts, such as The Cancer Genome Atlas (TCGA) project and the International Cancer Genome Consortium (ICGC), have profiled many thousands of human cancers, thereby producing a rich resource consisting of terabytes of somatic mutational data [40, 41]. This has catalysed a new field of research which has now been extended to the analysis of CN and SV signatures.

## The lexicon of copy number profiling: the devil is in the detail

**Figure 1:**
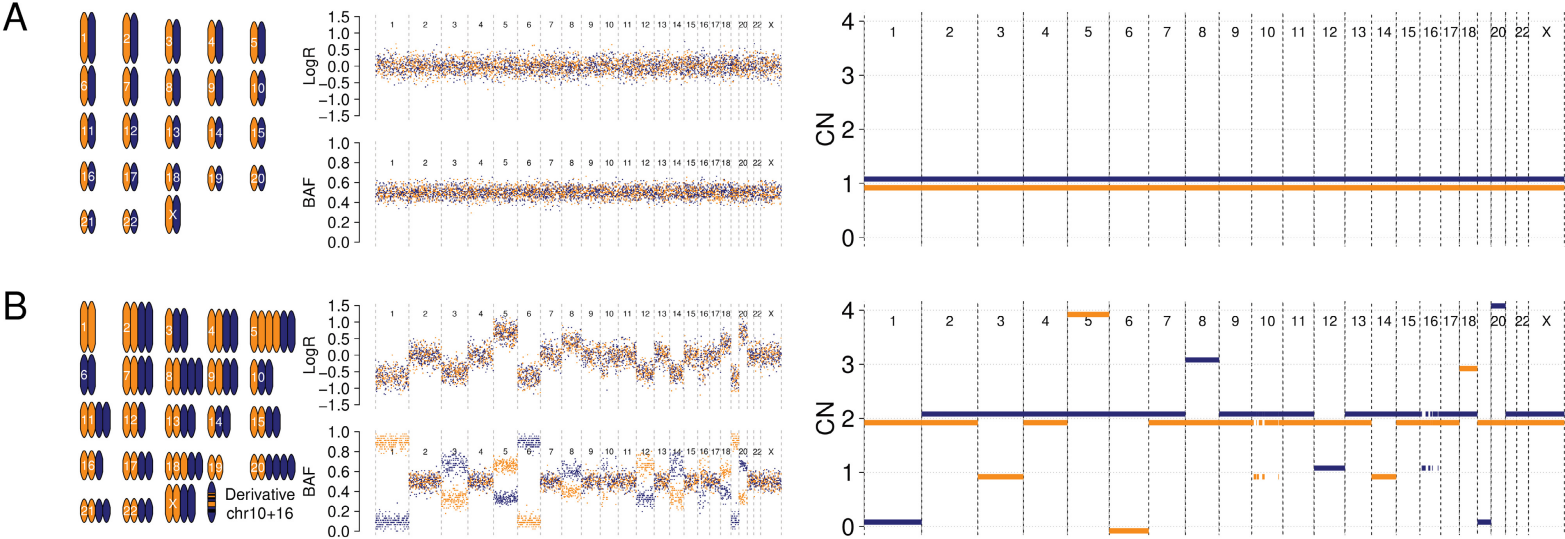
Examples of CN profiles derived from karyotyping and DNA sequencing. Mock karyotype (left), log*R* and BAF tracks (middle), and CN profile (right) for (A) a diploid and (B) an aneuploid genome. Maternal (blue) and paternal (orange) chromosomes are displayed separately and phased throughout. CN, copy number. The aneuploid genome is whole‐genome doubled (WGD) and includes losses prior to (chr1 + 6 + 19) and following (chr3 + 12 + 14) WGD, as well as gains prior to (chr5 + 20) and following (chr8 + 18) WGD. Additionally, a chromothripsis event after WGD involving both chr10 and chr16 is included.

The evaluation of chromosomes in cells through karyotyping is an established technique to evaluate aneuploidy either in the germline setting, e.g. to identify trisomy of chromosome 21 in Down's syndrome, or in the somatic setting, e.g. to reveal chromosomal changes in cancer [42]. This laboratory technique provides a global low‐resolution snapshot of CN aberrations that include large gains and losses of DNA, duplicated chromosomes, and translocations [43]. Higher throughput methods using comparative genomic hybridisation (CGH) arrays or single nucleotide polymorphism (SNP) arrays with vastly increased resolution allow for genome‐wide interrogation of CN down to the gene level on fresh, frozen, and even formalin‐fixed, paraffin‐embedded tissues, which has allowed for unprecedented insights into the CN landscape of cancer [44, 45]. The advantage of SNP arrays over CGH is that the genotyping of common SNPs enables the ability to infer not only the total CN by using the signal intensity (i.e. log*R*) across a region of the genome but also the ratio of reference and alternate allele intensities (B‐allele frequency) to elucidate the allele‐specific CN changes within the same region [46]. Next‐generation sequencing technologies, such as WES and WGS, can also be utilised to generate allele‐specific CN across the genome [47], by deriving log*R* from the sequencing depth and the B‐allele frequencies. This combination of log*R* and B‐allele frequency for a given sample is commonly referred to as the *copy number profile* of the sample (Figure 1). Multiple bioinformatic tools to derive CN profiles have been developed, ranging from relatively straightforward methods that utilise log*R* values to infer total CN [48], to methods that use both log*R* and BAF values to infer allele‐specific CN [46, 49, 50, 51], and even more sophisticated methods that infer not only clonal CN but also sub‐clonal CNAs through the application of haplotype phasing [25]. In addition, the choice of CN caller is often motivated by the utilised profiling platform, e.g. B‐allele frequencies are challenging to obtain from shallow WGS data (low sequencing coverage WGS), whereas haplotype phasing is challenging for non‐WGS data. Recent advances that combine information across multiple samples have improved CN profiling where samples are evolutionarily related, e.g. multi‐region sampling of a single tumour [52], while also paving the way for allele‐specific CN calling from shallow WGS and from single‐cell sequencing data [53].

For CN analysis, probably the most commonly used approach to decipher potential CN drivers from cancer genome samples is the Genomic Identification of Significant Targets in Cancer (GISTIC) tool [54]. Using this method, one can identify statistically significant regions of recurrent amplifications and deletions, in essence providing a method to identify potential CN driver mutations. Whilst extremely useful, the GISTIC method does not allow one to infer the mutational processes that may be generating those CN changes. In an analogous way to mutational signatures, the newly developed CN signature methods relate CN patterns to potential mutational processes.

## A deep look into the short history of copy number signatures

The field of mutational signature analysis has developed multiple methods based on non‐negative matrix factorisation, latent Dirichlet allocation, hierarchical Dirichlet processes, or other methods for data classification [15, 54, 55, 56] and tools [15, 57, 58, 59, 60, 61] to extract meaningful information from cancer genomes. An early study in 2018 on high‐grade serous ovarian cancer (OV) was one of the first to use such mathematical approaches to show the potential utility of a CN signature for prognostication [22]. OV is a genetically complex tumour characterised by *TP53* mutations, often associated with homologous recombination deficiency (HRD) (see below), breakage fusion bridge cycles – a pattern of replication‐associated genomic instability induced by telomere shortening, leading to amplifications and genomic rearrangements, and chromothripsis, which is a mutational process leading to clustered rearrangements that occur as a single event following the ‘shattering of a chromosome’ [55]. In their CN signature framework, Macintyre *et al* [22] designed a model to capture these salient genetic features of the OV genome through CN signatures derived from shallow WGS data (Table 1). Their analysis demonstrated the potential clinical utility of CN signatures by identifying a signature that was linked to poor prognosis in some patients. OV served as an ideal model upon which to build such a framework, as the endogenous DNA damage processes linked with the CN patterns were already well established in that cancer type [55, 56].

**Table 1:** Design of features for CN signature methods

Reference	Genomic feature	Biological process
Macintyre *et al* (2018) [22]	Breakpoint count per 10 Mb	Chromothripsis
	CN	
	CN change point	Breakage fusion bridge
	Breakpoint count per chromosome arm	Chromothripsis
	Length of chains of oscillating CN	Chromothripsis
	Segment size	HRD, chromothripsis
Wang *et al* (2021) [62]*	Minimum number of chromosomes with 50% CN altered (1 value)	Ploidy
	Number of events per chromosome	Genomic distribution of events
Steele *et al* (2019/2021) [23, 24]	LOH status	Mechanism‐agnostic^†^
	Total CN	
	Segment size	

* Additional features introduced to the method described in ref 22.

^†^ Patterns identified from mechanism‐agnostic approaches can be associated with various datasets *post hoc*; this allows for linking of multiple mechanism‐agnostic signatures with processes such as genome doubling, chromosomal instability, chromothripsis, HRD, and haploidisation [24].

The utility of signatures as biomarkers for treatment stratification in cancer is perhaps best exemplified by those developed for identifying HRD [22, 24]. Inactivating mutations in *BRCA1*, *BRCA2* or other HR pathway genes leave tumour cells unable to repair double‐strand breaks of the genome in a faithful manner, instead relying on error‐prone pathways such as non‐homologous end joining or microhomology‐mediated end joining [57, 58]. Antagonising this deficiency, either through inducing DNA damage (as for platinum‐based therapies) or through blocking compensatory repair pathways (as for PARP inhibition), is a promising therapeutic avenue [59]. Nevertheless, stratifying patients for these treatments remains an open challenge. The US Food and Drug Administration has approved the Myriad Genetics myChoice CDx® test, which combines identification of inactivating mutations of *BRCA1* or *BRCA2* along with specific CN‐based readouts of ‘genomic scars’ of HRD [60]. However, alterations of other HR pathway genes, or non‐genetic mechanisms, such as promoter methylation silencing [61], may abrogate the HR pathway but could be missed by the myChoice CDx® test. Similar CN‐based metrics inspired by the myChoice CDx® genomic scars have been developed [63]; however, the application of such metrics across different tumour types requires careful calibration [64]. Beyond CN genomic scars, signatures of single base substitutions, indels, and rearrangements have been associated with HRD tumours [15, 16, 21]. In an effort to generate a robust test for HRD, Davies *et al* built a predictive model for HRD – HRDetect – that incorporates SBS, indel, and rearrangement signatures, as well as CN genomic scars [65, 66], which holds great promise but is restricted to WGS data. Other methods that are dependent on whole‐genome sequencing [67] or that have removed the need for WGS data and are therefore applicable to targeted sequencing panels [68] have also been published. Additionally, the distinction between ongoing HRD and historic HRD needs to be properly assessed in tumours; historic HRD may leave the scars on the genome that indicate that the tumour is HR‐deficient, but the tumour may have reinstated HR through mechanisms such as *PTEN* mutation, or compensating mutations up‐ or down‐stream of the core HR pathway genes [69]. This distinction is important for patients, as ongoing HRD should be sensitive to PARP inhibition, whereas tumours that have reinstated HR may be resistant.

**Figure 2:**
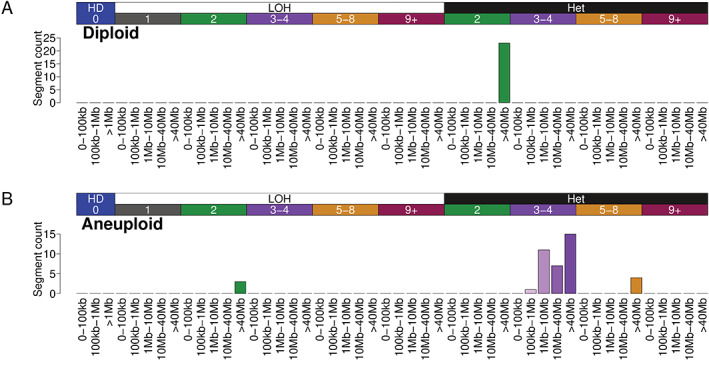
CN summary vectors for the two profiles displayed in Figure 1. (A) Diploid and (B) aneuploid, as per the summary methodology of Steele *et al* [24]. LOH status and total CN categories are displayed above the bar plots. Segment size categories are displayed below the bar charts.

**Figure 3:**
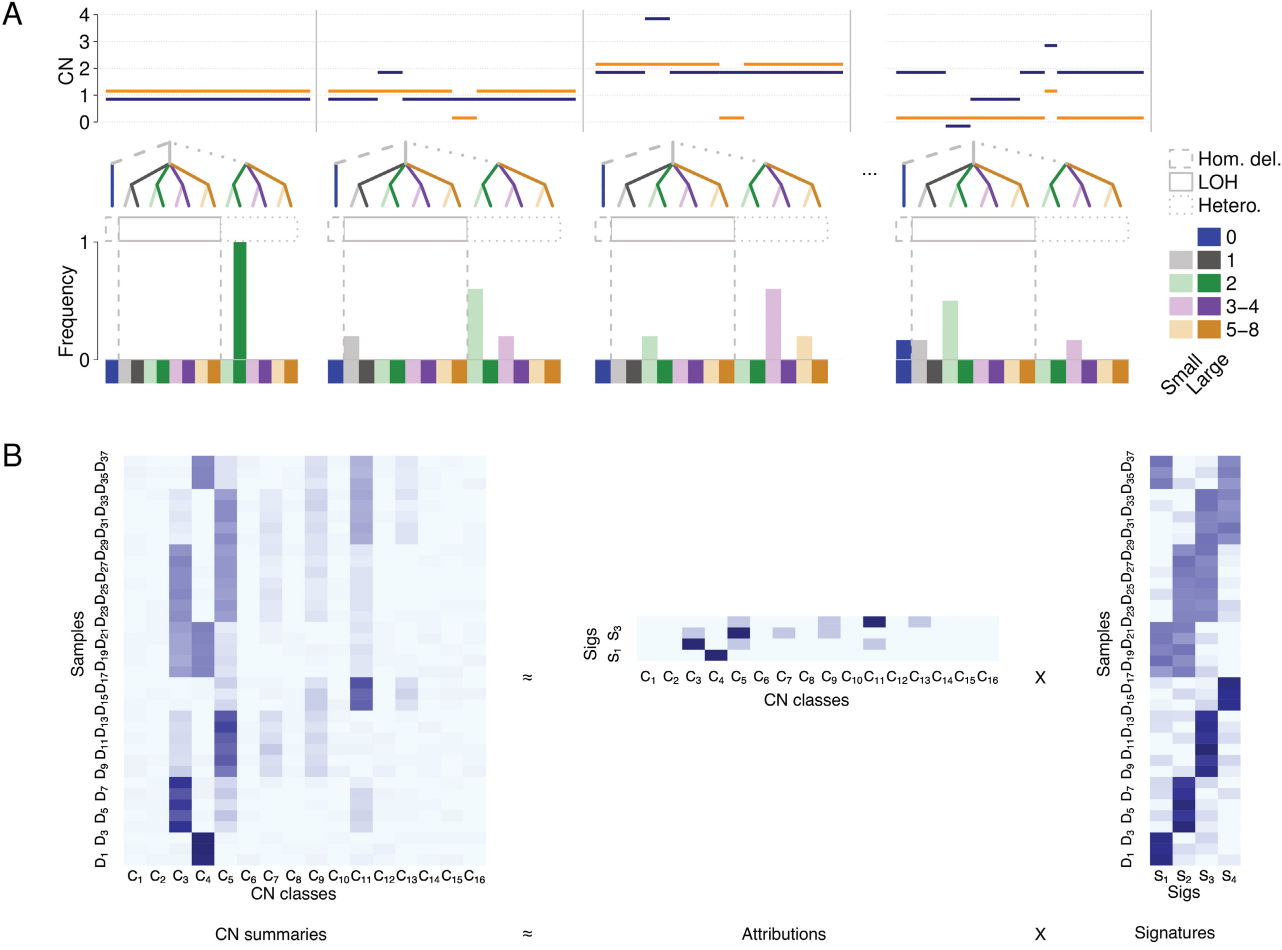
CN signature methodology (mechanism‐agnostic). (A) Allele‐specific CN profiles (top panels; *y*‐axis = allele‐specific CN, orange = minor CN, blue = major CN) have their segments classified by loss of heterozygosity (LOH) status, total CN, and segment size (middle panels; see legend in figure) to generate CN feature counts (bottom panels). Example profiles are shown for a diploid genome, a diploid genome with historic chromosomal instability, a genome double version of the previous genome, and a genome with extensive LOH (in order left to right). (B) CN feature counts for a dataset are combined into matrix of CN summaries (left), which are decomposed using non‐negative matrix factorisation, or another appropriate method, into a matrix of signature definitions (right) and a matrix of attributions of each of those signatures in the samples of the given dataset (middle).

In 2019, through a separate study of genomically complex undifferentiated soft tissue sarcomas (USARCs), where limited prior knowledge about the underlying mutational processes was known, Steele *et al* developed a ‘mechanism‐agnostic’ approach to summarise CN profiles into CN signatures [23]. This was achieved by categorising the segments of an allele‐specific CN profile by fundamental aspects of CN into a CN summary vector, which included loss of heterozygosity (LOH) status, total CN state, and segment size (Figure 2A,B). Once a set of CN summaries is obtained (Figure 3A), the matrix of all summaries can be decomposed into a matrix of signature definitions and a matrix of signature attributions (Figure 3B) using non‐negative matrix factorisation. The set of signatures and their attributions can then be used to infer the potential biological relevance of the signatures in question through associations with relevant data such as driver mutations, chromothripsis, whole‐genome doubled (WGD) status, or even orthogonal information such as transcriptome profiling [22, 23, 24, 62, 70]. In USARCs, seven distinctive signatures were identified which were linked to biological processes including successive whole‐genome doublings, genome‐wide LOH events, and chromothripsis [23]. We were able to integrate these signatures with other genomic data to elucidate multiple evolutionary routes through which USARCs potentially develop, demonstrating the utility of CN signatures for understanding the evolutionary history of cancer genomes. Recently, Steele *et al* expanded this work to ~10 000 cancers to develop a pan‐cancer set of 21 CN signatures [24], with new signatures linked to HRD, extrachromosomal circular DNA formation, and haploidisation. Moreover, this work provided a further refinement in the evolutionary relationship between CN signatures and their role in tumour history.

The two signature methods described above broadly divide the field into two major classes: those that have CN features designed specifically to capture previously known biological processes [22, 62, 70] and those designed to be mechanism‐agnostic [23, 24] (Table 1). Importantly, patterns identified from mechanism‐agnostic approaches can be subsequently associated with various datasets *post hoc*; this has allowed the linking of multiple mechanism‐agnostic signatures with processes such as genome doubling, chromosomal instability, chromothripsis, HRD, and haploidisation [24]. While mechanism‐agnostic approaches may not capture all known biological processes, they can reveal previously unappreciated molecular mechanisms [24]. In contrast, while CN features designed to capture a particular set of biological processes will capture these processes, they would rarely elucidate previously unknown molecular mechanisms.

Macintyre *et al* [22] designed a set of CN features that would each tag a given process known to generate CN profiles with distinctive values for those features, e.g. classical chromothripsis will generate many breakpoints with long oscillating CN runs of short segment size [71, 72] (Table 2). In addition to the studies outlined above, a set of other CN signature analyses were also performed, with most of them focusing on detecting specific biological processes. For example, the above set of features was expanded by Wang *et al* [62] to include metrics of global CN aberration extent and local CN aberration extent per chromosome (Table 2). To convert the feature space into counts that are suitable for decomposition, Macintyre *et al* utilised mixture models to categorise the feature distributions (Table 2). However, this mixture model approach may lead to different categories between different datasets or profiling platforms, causing difficulties when comparing results across different studies. To rectify this, Wang *et al* instead generated manual categories for each feature type, allowing for the same categories to be used across studies. A further study in multiple myeloma took a hybrid approach where categories for most features were determined using mixture models, but categories for CN were manually designed, specifically to fit the known biology of the malignancy [70].

**Table 2:** Overview of published CN signature methods

	Macintyre *et al* (2018) [22]	Wang *et al* (2021) [62]	Maclachlan *et al* (2021) [70]	Steele *et al* (2019) [23]	Steele *et al* (2021) [24]
Tumour	Ovarian	Prostate	Multiple myeloma	USARC	Pan‐cancer
Number of samples	385	1003	752	52	11 210
Number of signatures	7	5	5	7	21
Mechanism	Designed	Designed	Designed	Agnostic	Agnostic
Summary method	Mixture models	Manual categories	Hybrid (manual category – total CN)	Manual categories	Manual categories
Decomposition method	NMF	NMF	HDP	NMF	NMF
Number of components	36	80	28	40	48
Platforms	Shallow WGS	Exome	Shallow WGS	Deep WGS	SNP6 microarray Exome sequencing Deep WGS Shallow WGS Reduced representation bisulphite sequencing
Allele‐specific	No	No	No	Yes	Yes
Genomic information	None	Chromosomal counts	None	None	*Post hoc* mapping
Included segments	All	All	All	All	All
Ploidy sensitive	Yes	Yes	Yes	Yes	Yes
Software	Custom code	Sigminer	Custom code	Custom code	SigProfiler

NMF, non‐negative matrix factorisation; HDP, hierarchical Dirichlet process.

## Biological considerations of copy number signatures

Multiple studies have now demonstrated the utility of CN signatures to predict the prognosis of patients, both in a single‐tumour [22, 62, 70] and in a pan‐cancer context [24] (Table 2). Synthesis of the results to date suggests that patients with tumours that exhibit patterns indicating an amplicon or chromothripsis‐like event have poor survival [24, 70]. This corroborates previous findings of poor survival of patients with chromothriptic tumours [73] or with extra‐chromosomal circular DNA [74].

**Figure 4:**
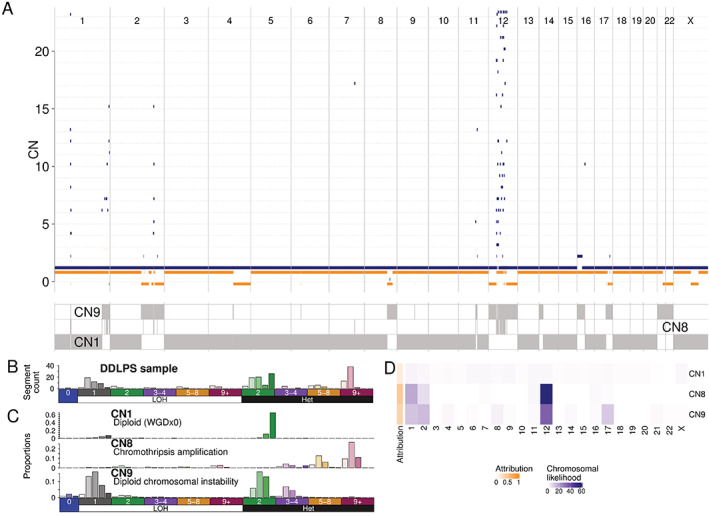
*Post hoc* mapping of CN signatures to the genome. (A) CN profile of a dedifferentiated liposarcoma (DDLPS) with characteristic amplification of chr12 including MDM2. Orange indicates minor CN; blue indicates major CN. The bottom panel indicates regions of the genome that have been mapped to a signature (in grey); regions of the genome unattributed to a signature are in white. (B) CN summary vector for the DDLPS in A. See legend of Figure 2 for the ordering of CN classes. (C) Signature definitions for the three signatures attributed to this sample. CN classes from B can be explained by each of the three signatures. (D) Heatmap of the likelihood of signatures (*y*‐axis) explaining the segments in a genomic bin of the window (blue), where here the bins are the chromosomes of the human genome (*x*‐axis). Global attributions of the three signatures to this sample are coloured in orange. Signature 8, a signature associated with chromothripsis amplification, is attributed to chromosomes 12, 1, and 2, due to the highly segmented patterns seen on those chromosomes. Once the chromosomal likelihoods are obtained, the maximum likelihood signature for each segment based on its CN class can be assigned, giving the assignments shown in the bottom panel of A.

The relative simplicity of the signature encoding in mechanism‐agnostic approaches enables the mapping of signatures back to the genome, reconstituting the genomic context of the CN signatures [24], thereby providing information about the processes that generate the CN event. One notable example is the identification of chromothripsis signatures associated with oncogenic *MDM2* amplification in dedifferentiated liposarcoma (Figure 4) consistent with the recently described complex SV coupled with amplification known as *tyfonas* [30]. This result highlights the importance of genomic context when interpreting CN signatures, especially with the understanding that the CN landscape of a cancer cell is heavily shaped by positive and/or negative selection [75]. Beyond identifying known tumour suppressor genes/oncogenes using CN signatures, there may be further utility in discovering novel cancer driver genes, particularly in cohorts of rare or understudied tumours. The recurrence of specific signatures in regions of the genomes, including distinct recurrence patterns in individual tumour types [24], reflects the strong selective pressures acting on CNAs more broadly. This mirrors known phenomena such as recurrent aneuploidies seen in individual tumour types [76], driven through the interplay between loss of tumour suppressor genes, gain of oncogenes, and retention of essential genes [75]. In contrast, the vast majority of SBS mutations seen in a tumour genome are passenger events [77], and possibly have little effect on selection. This distinction suggests that SBS signatures provide a more unbiased window into the processes that generated them, whereas CN profiles have a much stronger inherent bias. This selection pressure has been directly observed in colon organoids, where daughter cells of chromosomal mis‐segregation events have a high probability of subsequent cell death [78], whereas the same is not true of the vast majority of SBS mutations [27].

## Future directions

**Figure 5:**
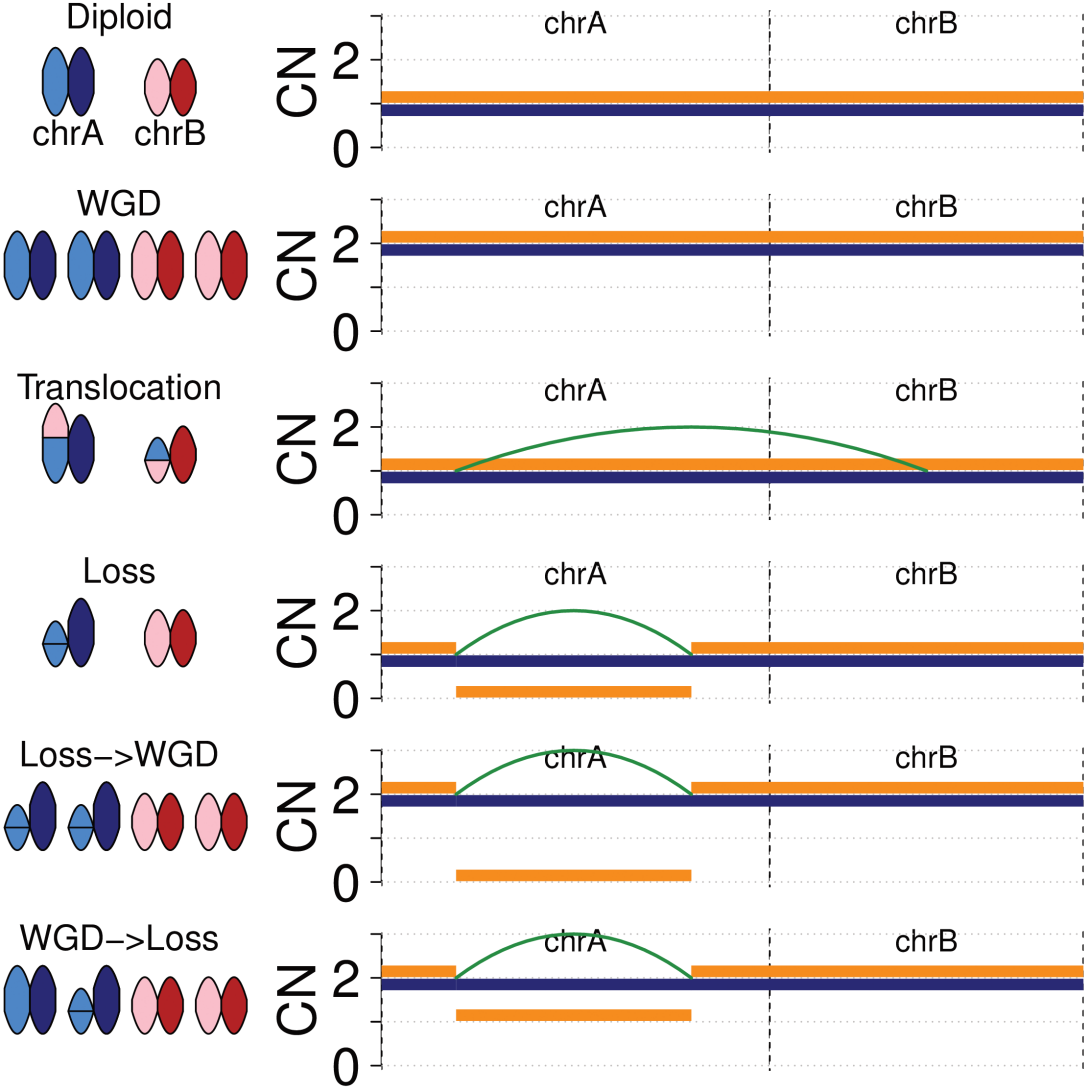
Schematic diagram of hypothetical genome configurations for two chromosomes (left; blue = chromosome A, red = chromosome B) and their associated CN and rearrangement profiles (*y*‐axis = allele‐specific CN, *x*‐axis = genome, orange = minor CN, blue = major CN, green = rearrangement). Specific structural alterations to the genome may lead to only CN alterations (whole‐genome doubling; WGD), only rearrangements (translocation), or a combination of both (loss). Further, the consequences of the same structural alterations (WGD and a loss) may depend on the order in which those structural alterations occurred; a loss followed by WGD (Loss → WGD) will lead to loss of heterozygosity for the lost segment, whereas the reverse ordering (WGD → Loss) will retain heterozygosity for that segment, which may be important when assessing second hits to tumour suppressor genes.

CNAs and rearrangements represent two distinct but related consequences of structural alteration to the genome (Figure 5). As a result, it is likely that signatures that incorporate both rearrangements and CN profiles would more fully describe the consequences of structural phenomena. As an example, a whole‐genome doubling event will be ‘silent’ when viewed through rearrangement but will be observed through CN data. In contrast, a balanced translocation or an inversion may be ‘silent’ within a CN profile but will be observed through rearrangement data. This may help to distinguish different molecular events, e.g. chromothripsis that has occurred on single versus on multiple chromosomes and may further refine patient stratification.

A distinction between CN changes and single base substitutions is that every substitution can be examined as a single independent event, barring local clusters of hypermutation [28] or violations of the infinite sites model [79, 80]. In contrast, every segment in a CN profile cannot be evaluated as a single or as an independent event, as these CN events may occur simultaneously [81]. As a result, the interpretation of a CN profile is limited, in that you can only necessarily observe the end stage of a process that has generated the patterns. To exploit the potential of CN signatures for evolutionary studies, it may be valuable to perform sub‐clonal CN reconstruction to discover the clonal composition of the tumour, and hence map CN events onto the tumour's evolutionary tree. In this way, it may be possible to develop a new generation of signatures of CNAs (event‐level signatures) that may more accurately reflect the signatures of the observed CN profiles (end‐state signatures). For SBS data, the difference between event‐level and end‐state‐level is minimal [28], whereas for CN data it may be substantial.

Lastly, considerable effort has been expended to validate that signatures of small mutational events correspond to real biological processes [26, 27, 82, 83]. Similar efforts will be required for CN signatures; however, the experimental strategies for inducing CNAs and/or processes are less deterministic and may necessitate careful experimental design. As a further complication, the CN profile generated from an individual event will depend on the previous history of the tumour genome, e.g. a loss before and after genome doubling will lead to loss or retention of heterozygosity, respectively (Figure 5). Despite these challenges, the emerging evidence suggests that CN signatures represent a fertile research area to understand cancer pathogenesis and can be utilised as a robust approach for prognostication and, in some cases, for therapeutic stratification of cancer patients.

## Author contributions statement

The review was conceived, designed, written, and edited by CDS, NP, and LBA.
